# Disruption of Early Tumor Necrosis Factor Alpha Signaling Prevents Classical Activation of Dendritic Cells in Lung-Associated Lymph Nodes and Development of Protective Immunity against Cryptococcal Infection

**DOI:** 10.1128/mBio.00510-16

**Published:** 2016-07-12

**Authors:** Jintao Xu, Alison J. Eastman, Adam Flaczyk, Lori M. Neal, Guolei Zhao, Jacob Carolan, Antoni N. Malachowski, Valerie R. Stolberg, Mohammed Yosri, Stephen W. Chensue, Jeffrey L. Curtis, John J. Osterholzer, Michal A. Olszewski

**Affiliations:** a Division of Pulmonary and Critical Care Medicine, Department of Internal Medicine, University of Michigan Health System, Ann Arbor, Michigan, USA; b Graduate Program in Immunology, University of Michigan Health System, Ann Arbor, Michigan, USA; c Pulmonary Section, Medical Service, Ann Arbor VA Health System, Department of Veterans Affairs Health System, Ann Arbor, Michigan, USA

## Abstract

Anti-tumor necrosis factor alpha (anti-TNF-α) therapies have been increasingly used to treat inflammatory diseases and are associated with increased risk of invasive fungal infections, including *Cryptococcus neoformans* infection. Using a mouse model of cryptococcal infection, we investigated the mechanism by which disruption of early TNF-α signaling results in the development of nonprotective immunity against *C. neoformans*. We found that transient depletion of TNF-α inhibited pulmonary fungal clearance and enhanced extrapulmonary dissemination of *C. neoformans* during the adaptive phase of the immune response. Higher fungal burdens in TNF-α-depleted mice were accompanied by markedly impaired Th1 and Th17 responses in the infected lungs. Furthermore, early TNF-α depletion also resulted in disrupted transcriptional initiation of the Th17 polarization program and subsequent upregulation of Th1 genes in CD4^+^ T cells in the lung-associated lymph nodes (LALN) of *C. neoformans*-infected mice. These defects in LALN T cell responses were preceded by a dramatic shift from a classical toward an alternative activation of dendritic cells (DC) in the LALN of TNF-α-depleted mice. Taken together, our results indicate that early TNF-α signaling is required for optimal DC activation, and the initial Th17 response followed by Th1 transcriptional prepolarization of T cells in the LALN, which further drives the development of protective immunity against cryptococcal infection in the lungs. Thus, administration of anti-TNF-α may introduce a particularly greater risk for newly acquired fungal infections that require generation of protective Th1/Th17 responses for their containment and clearance.

## IMPORTANCE

Increased susceptibility to invasive fungal infections in patients on anti-TNF-α therapies underlines the need for understanding the cellular effects of TNF-α signaling in promoting protective immunity to fungal pathogens. Here, we demonstrate that early TNF-α signaling is required for classical activation and accumulation of DC in LALN of *C. neoformans*-infected mice. Subsequent transcriptional initiation of Th17 followed by Th1 programming in LALN results in pulmonary accumulation of gamma interferon- and interleukin-17A-producing T cells and effective fungal clearance. All of these crucial steps are severely impaired in mice that undergo anti-TNF-α treatment, consistent with their inability to clear *C. neoformans*. This study identified critical interactions between cells of the innate immune system (DC), the emerging T cell responses, and cytokine networks with a central role for TNF-α which orchestrate the development of the immune protection against cryptococcal infection. This information will be important in aiding development and understanding the potential side effects of immunotherapies.

## INTRODUCTION

While serving as an important inflammatory mediator, tumor necrosis factor alpha (TNF-α) can induce many detrimental effects in a broad range of diseases, such as sepsis, cancer, and autoimmune disorders (1). Anti-TNF-α therapy has been widely used for treatment of these diseases during the past decades. However, more recently it has been recognized that TNF-α blockade significantly increases the risk for invasive fungal infections, including *Cryptococcus neoformans* infection (2, 3). Thus, better knowledge of how TNF-α regulates immune responses during fungal infections is needed to understand and offset the risks associated with therapies blocking TNF-α signaling.

*C. neoformans* triggers infection in patients with various immunocompromised states. After entering the host through the respiratory tract, it can disseminate to extrapulmonary organs, including the central nervous system (4–7). Murine models have shown that protective anticryptococcal immunity depends on the generation of T cell-mediated immune responses (8, 9). Strong Th1/Th17 responses promote the effective containment and elimination of *C. neoformans* (10–12), while the Th2 response impairs fungal clearance (13–15). Further, TNF-α signaling has been shown to promote protective immune responses and subsequent fungal clearance during cryptococcal infection with the moderately virulent strain 24067 (16). Transient TNF-α depletion in mice at the time of infection with *C. neoformans* resulted in a temporary decrease in interleukin-12 (IL-12) and gamma interferon (IFN-γ) production during the afferent phase, followed by recovery of their production during the efferent phase (17, 18). Interestingly, this recovery of protective cytokine production occurred without restoration of fungal clearance (17, 18), suggesting the possibility of a lasting defect in T cell polarization and/or activation. Thus, the effect of early TNF-α depletion on the polarization/activation of CD4^+^ T cells during cryptococcal infection needs to be accurately assessed.

Dendritic cells (DC) play a predominant role in presenting antigen and directing T cell polarization (19, 20). The immature status of DC has been previously suggested to account for the immune dysregulation in *C. neoformans*-infected mice following TNF-α depletion (18). However, more recent studies with other antigens and/or pathogens suggest that DC can become either classically or alternatively activated, which then directs the subsequent type of T cell polarization (21, 22). Classically activated DC (DC1) show robust production of Th1/Th17-polarizing cytokines along with high surface expression of major histocompatibility complex class II (MHC-II) and costimulatory molecules (23). Robust expression of all of these factors is implicated in the development of protective Th1/Th17 responses (23). In contrast, alternatively activated DC (DC2), which are implicated in the generation of a Th2 immune bias, show diminished expression of DC1 markers, enhanced induction of Th2-polarizing cytokines, and upregulation of markers consistent with those found in alternatively activated (M2) macrophages (21, 24). The effects of early TNF-α signaling and/or its disruption on the DC polarization profile in the lungs and lung-associated lymph nodes (LALN) during cryptococcal infection have not been studied.

Additionally, it remains unknown to what degree T cell programming occurs in the LALN during cryptococcal infection. For instance, Lindel et al. presented evidence that, in contrast to T cells isolated from lungs, LALN T cells did not produce IFN-γ in response to cryptococcal antigen (25). Furthermore, Wiesner et al. showed that lymphoid organ priming was not required for pulmonary Th2 cell accumulation during cryptococcal infection (26). Thus, further investigations of the roles of cellular and molecular interactions in LALN during development of protective versus nonprotective anticryptococcal responses are needed.

Here, we report that early TNF-α signaling is responsible for the acquisition of a DC1 phenotype by LALN DC, which optimally activates and polarizes CD4^+^ T cells to the Th17 and Th1 lineage in the LALN during the afferent phase of cryptococcal infection. Subsequent recruitment of Th1 and Th17 cell subsets producing IFN-γ and IL-17A in *C. neoformans*-infected lungs correlates with progressive fungal clearance. In contrast, the disruption of early TNF-α signaling results in the development of a DC2 phenotype by LALN DC, which ultimately leads to the development of nonprotective immune responses to *C. neoformans* infection.

## RESULTS

### Early TNF-α depletion diminishes protective Th1- and Th17-biased immune responses in *C. neoformans*-infected mice.

**FIG 1:**
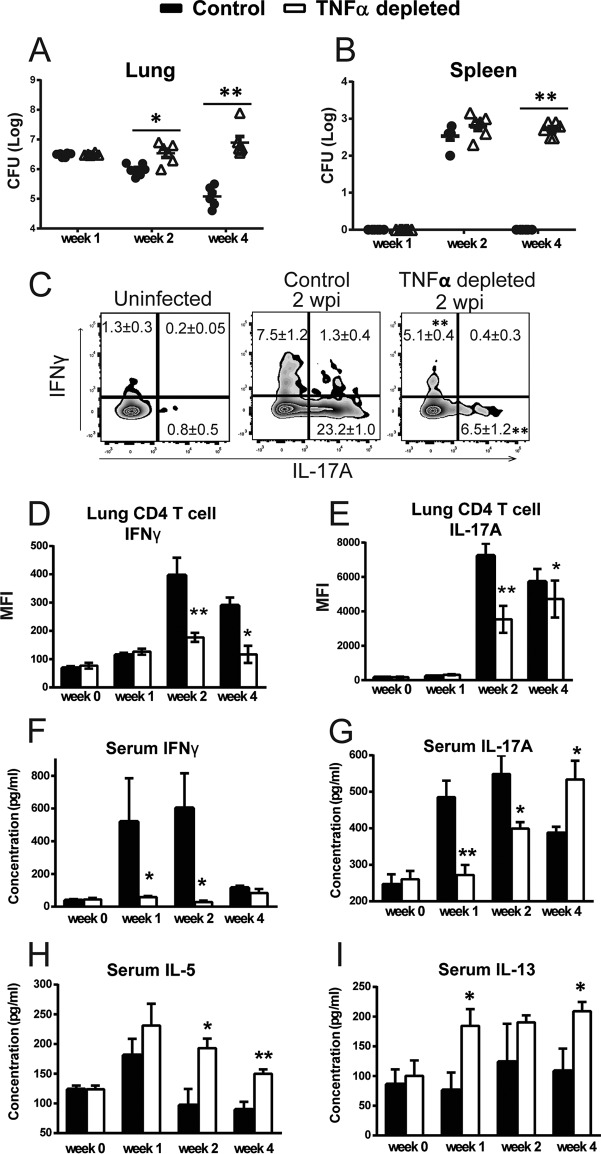
Neutralization of TNF-α results for diminished Th1- and Th17-biased immune responses in *C. neoformans*-infected mice. Mice were infected intratracheally with 10^4^ *C. neoformans* 52D and treated with anti-TNF-α antibody or an isotype control at the time of infection. (A and B) Fungal burdens in the lungs (A) and spleens (B) were higher during the efferent phase of infection in TNF-α-depleted mice than in the control mice. (C) Flow cytometry analysis detected diminished frequencies of IFN-γ- and IL-17A-positive CD4^+^ T cells from the lungs in TNF-α-depleted mice compared to the control mice. (D and E) Bar graphs represent the mean fluorescence intensity of IFN-γ-positive (D) and IL-17A-positive (E) CD4^+^ T cells at 0, 1, 2, and 4 wpi. (F and G) Serum cytokine analysis revealed significantly lower levels of IFN-γ (F) and IL-17A (G) in TNF-α-depleted mice than in control mice. (H and I) Significantly higher levels of IL-5 (H) and IL-13 (I) in the serum were detected at 1, 2, or 4 wpi in TNF-α-depleted mice than in the control mice. Results represent means ± SEM pooled from two separate matched experiments (*n* ≥ 6 mice for each time point). A two-way ANOVA with a Bonferroni posttest was used for comparisons of all individual means. *, *P* < 0.05; **, *P* < 0.01 compared with control mice.

The precise effect of TNF-α in T cell polarization in cryptococcal infection remains unknown; thus, our first objective was to determine whether TNF-α is required for the development of a protective Th1/Th17 bias in *C. neoformans*-infected mice. CBA/J mice were infected intratracheally with 10^4^ *C. neoformans* and injected with a single dose of isotype or anti-TNF-α neutralizing antibody at the time of infection, as described previously (17). Fungal burdens in the lung and spleen were compared, with concurrent analysis of cytokine production by pulmonary T cells and systemic (serum) cytokine levels. We observed significantly higher fungal burdens in the lungs (2 and 4 weeks postinfection [wpi]) and spleen (4 wpi) of anti-TNF-α-treated mice than in isotype-treated control mice, consistent with timing of the effector phase of T cell-mediated responses (Fig. 1A and B). These data are consistent with published work that reported that TNF-α depletion impaired fungal control during *C. neoformans* infection (16, 18). Impaired fungal clearance in TNF-α-depleted mice was associated with significant reductions in the frequency and intensity of IFN-γ- and IL-17A-producing pulmonary CD4^+^ T cells at 2 wpi and 4 wpi compared with isotype control-treated mice, as analyzed by intracellular flow cytometry (Fig. 1C, D, and E). Consistently, mice subjected to early TNF-α depletion had significantly diminished serum concentrations of IFN-γ and IL-17A at 1 and 2 wpi relative to control mice (Fig. 1F and G). In contrast, early TNF-α depletion resulted in significantly higher serum concentrations of Th2 cytokines IL-5 (2 and 4 wpi) and IL-13 (1 and 4 wpi) (Fig. 1H and I). Collectively, these findings show that early TNF-α signaling is required for the local development of Th1/Th17 CD4^+^ T cell polarization in the lungs and a protective systemic immune response during cryptococcal infection.

### Early TNF-α depletion impairs Th1 and Th17 polarization at the transcriptional level and CD4^+^ T cell activation in the LALN of *C. neoformans*-infected mice.

**FIG 2:**
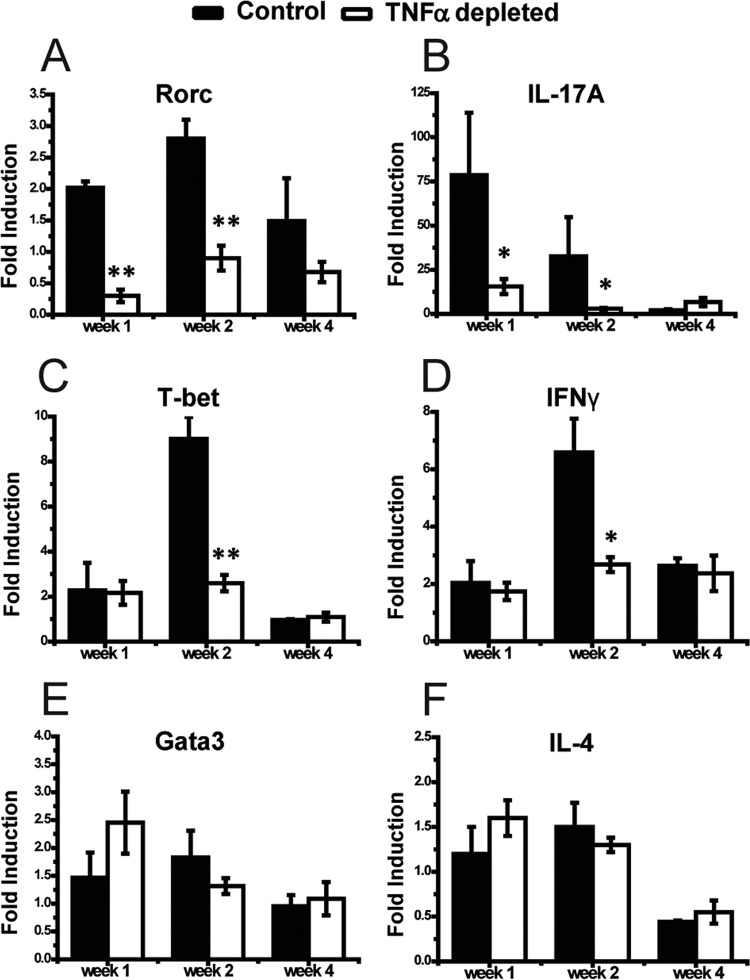
Neutralization of TNF-α affects Th1/Th17 polarization of CD4^+^ T cells in the LALN of *C. neoformans*-infected mice. Leukocytes were isolated from the LALN of infected mice and helper T cells were sorted using anti-CD4 magnetic beads. Total RNA was isolated and analyzed by RT-qPCR. The mRNA expression levels of Th17/Th1/Th2 transcriptional regulators Rorc, T-bet, and Gata3, respectively (A, C, and E) and Th17/Th1/Th2-driving cytokines IL-17A, IFN-γ, and IL-4, respectively (B, D, and F) in the CD4^+^ T cells were analyzed. Note that there was a significant suppression of Th17 and Th1 markers, but not Th2 markers, in the TNF-α-depleted mice compared to the control mice. Results represent means ± SEM pooled from two separate matched experiments (*n* ≥ 6 mice for each time point). A two-way ANOVA with a Bonferroni posttest was used for comparison of all individual means. *, *P* < 0.05; **, *P* < 0.01 compared with control mice.

Having determined that early TNF-α signaling is required for skewing pulmonary and systemic adaptive immune responses in favor of protective Th1/Th17 immune polarization, we next sought to determine whether early TNF-α signaling impacted T cell polarization and (or) activation at a location further “upstream” of the lungs in the LALN. We first performed a kinetic analysis of mRNA expression for cytokines and transcription factors associated with Th polarization by using purified LALN CD4^+^ cells obtained from mice treated with anti-TNF-α or isotype control antibody. We observed significant reductions in mRNA expression of Rorc (a Th17 transcriptional regulator) and IL-17A (1 and 2 wpi) (Fig. 2A and B), followed by suppressed expression of the Th1-driving transcription factor T-bet and cytokine IFN-γ (2 wpi) (Fig. 2C and D) in mice treated with anti-TNF-α antibody compared to isotype control-treated mice. In contrast, early TNF-α blockade did not affect the expression of the Th2 transcriptional factor Gata3 (Fig. 2E) and Th2 cytokines IL-4/IL-5 (Fig. 2F and data not shown) at any of the studied time points. Finally, we evaluated the expression of FoxP3 by CD4^+^ T cells in the LALN at 1, 2, and 4 wpi to determine if TNF-α showed a significant effect on Treg populations in LALN. We found that FoxP3 expression was not affected by TNF-α depletion at any of these time points (data not shown).

**FIG 3:**
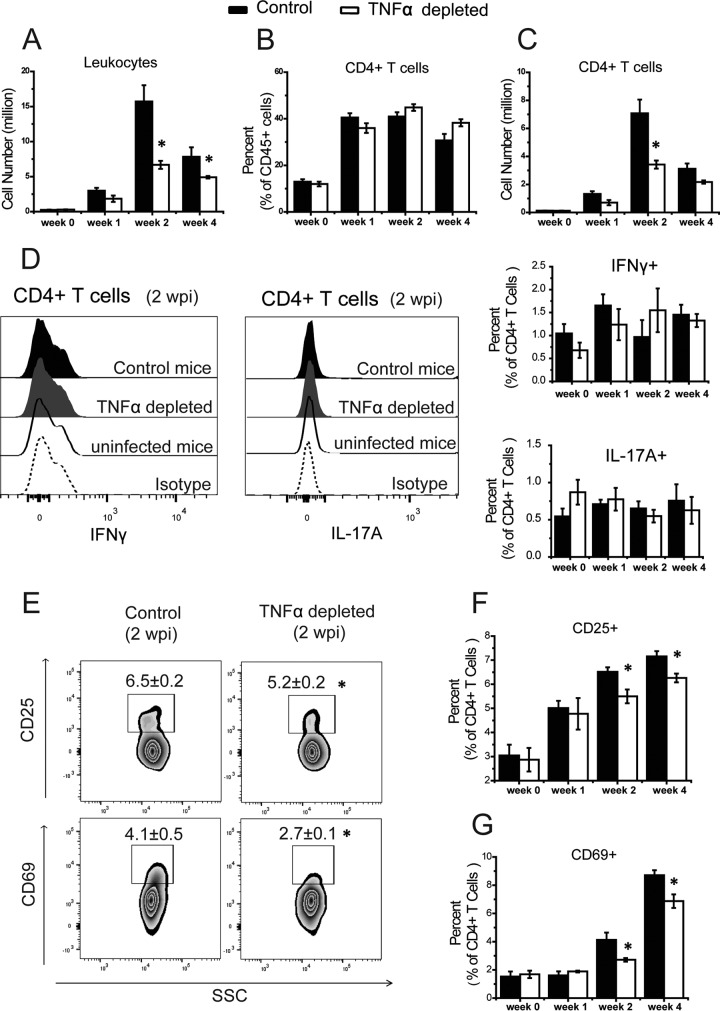
Neutralization of TNF-α results in impaired expansion and activation of CD4^+^ T cells in the LALN of *C. neoformans*-infected mice. Leukocytes were isolated from LALN of TNF-α-depleted and control mice and analyzed by flow cytometry. (A) Total numbers of leukocytes were reduced in the anti-TNF-α-treated mice compared to control mice. (B and C) A similar frequency (B) but lower total number (C) of CD4^+^ T cells in anti-TNF-α-treated mice relative to levels in control mice were detected. (D) Flow cytometry analysis showed that CD4^+^ T cells from LALN do not produce IFN-γ or IL-17A at the protein level in either TNF-α-depleted or control groups at 2 wpi. The bar graphs on the right present the frequency of IFN-γ- and IL-17A-producing CD4^+^ T cells in the LALN of infected mice. (E) T cell activation analyzed by flow cytometry revealed lower surface expression of the activation markers CD25 and CD69 by CD4^+^ T cells at 2 and 4 wpi in TNF-α-depleted and control mice. (F and G) The bar graphs present the frequency of CD25-positive (F) and CD69-positive (G) cells as percentages of total CD4^+^ T cells. Results represent means ± SEM pooled from two separate matched experiments (*n* ≥ 6 mice for each time point). A two-way ANOVA with a Bonferroni posttest was used for comparison of all individual means. *, *P* < 0.05 compared with control mice.

We further assessed the level of expansion, activation status, and cytokine production by CD4^+^ T cells in the LALN of *C. neoformans*-infected mice by using flow cytometry. We found that early depletion of TNF-α was associated with reduced LALN leukocyte numbers at 2 and 4 wpi (Fig. 3A). Although the percentage of CD4^+^ T cells did not differ between treatment groups (Fig. 3B), we identified a lower total number of CD4^+^ T cells at 2 wpi in ΤΝF-α-depleted mice than in isotype control-treated mice (Fig. 3C). Intracellular flow cytometry analysis showed that in contrast with upregulation of mRNA expression for IFN-γ or IL-17A, the LALN T cells in both control and TNF-α-depleted mice were not producing these cytokines (Fig. 3D), suggesting that the T cells became prepolarized at the transcriptional level in the LALN but required restimulation at the infected lungs for the production of cytokine proteins. However, T cell activation analyzed by surface expression of CD25 and CD69 markers showed a significantly lower percentage of CD4^+^ T cells expressing these markers at 2 and 4 wpi in TNF-α-depleted mice compared to isotype control-treated mice (Fig. 3E, F, and G). Collectively, these data show that disruption of early TNF-α signaling in mice infected with *C. neoformans* profoundly inhibits early events associated with the Th1/Th17 CD4^+^ T cell programming, expansion, and activation in the LALN.

### Early TNF-α depletion impairs dendritic cell accumulation but does not affect their spatial relationships with CD4^+^ T cells in the LALN of *C. neoformans*-infected mice.

**FIG 4:**
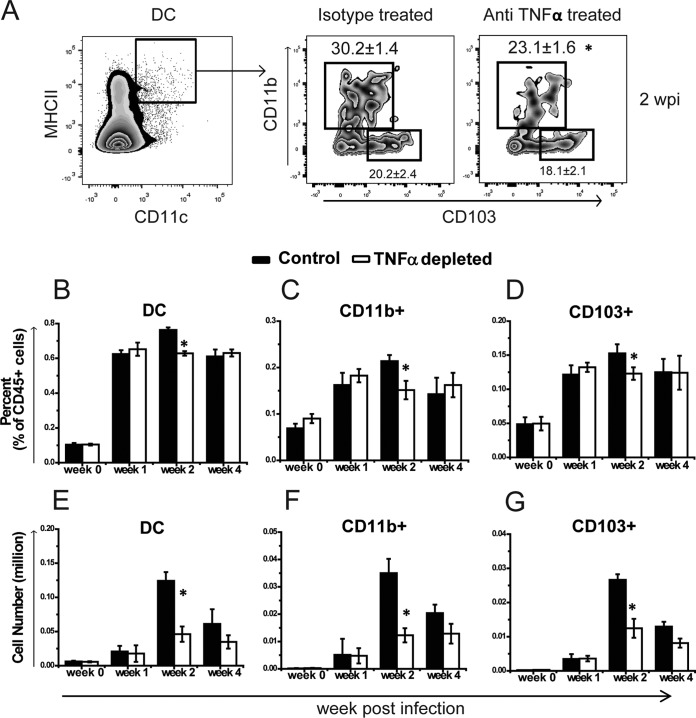
Neutralization of TNF-α impairs DC accumulation in the LALN of *C. neoformans*-infected mice. Leukocytes were isolated from the LALN of TNF-α-depleted or control mice at 0, 1, 2, and 4 wpi. (A) Representative flow cytometric plots show the gating scheme for DC (CD11c^+^ MHC-II^hi^), CD11b^+^ DC, and CD103^+^ DC at 2 wpi in TNF-α-depleted mice compared to the control mice. (B to D) Frequency of gated DC (B), CD11b^+^ DC (C), and CD103^+^ DC (D) decreased in the anti-TNF-α-treated mice compared to the control mice at 2 wpi. (E to G) Lower total numbers of DC (E), CD11b^+^ (F), and CD103^+^ (G) subsets were also detected in TNF-α-depleted mice than control mice. Results represent means ± SEM pooled from three separate matched experiments (*n* ≥ 10 mice for each time point). A two-way ANOVA with a Bonferroni posttest was used for comparison of all individual means. *, *P* < 0.05 compared with control mice.

Disrupted T cell polarization and activation in the LALN of *C. neoformans*-infected mice following early TNF-α blockade suggested a defect in the interactions of antigen-presenting cells (APCs) with naive T cells in the LALN. Prior studies had shown that DC take up fungal antigen in the lungs (27), traffic to the LALN, and orchestrate the subsequent adaptive T cell response (20, 28, 29). We therefore examined whether the defects in T cell polarization, expansion, and activation observed in TNF-α-depleted mice were associated with changes in DC accumulation and immunophenotype in the LALN. We first assessed the effects of early TNF-α depletion on the frequency and total number of DC (CD11c^+^ MHC-II^hi^) and two DC subsets: CD11b^+^ DC (CD11b^+^ CD103^−^), as well as a small proportion of the conventional CD11b^+^ CD103^−^ DC which acquired a CD11b^+^ CD103^+^ phenotype postinfection (30) and CD103^+^ DC (CD11b^−^ CD103^+^). Both of these DC subsets can take up antigens, migrate to the LALN, and prime T cells (31). We found that early depletion of TNF-α resulted in an overall reduction in relative percentages and total numbers of DC, as well as reductions in percentages and total numbers of both CD11b^+^ and CD103^+^ DC subsets at 2 wpi compared to the isotype control-treated mice (Fig. 4A to G). These data indicated that early TNF-α signaling contributes to optimal DC accumulation in the LALN during *C. neoformans* infection.

**FIG 5:**
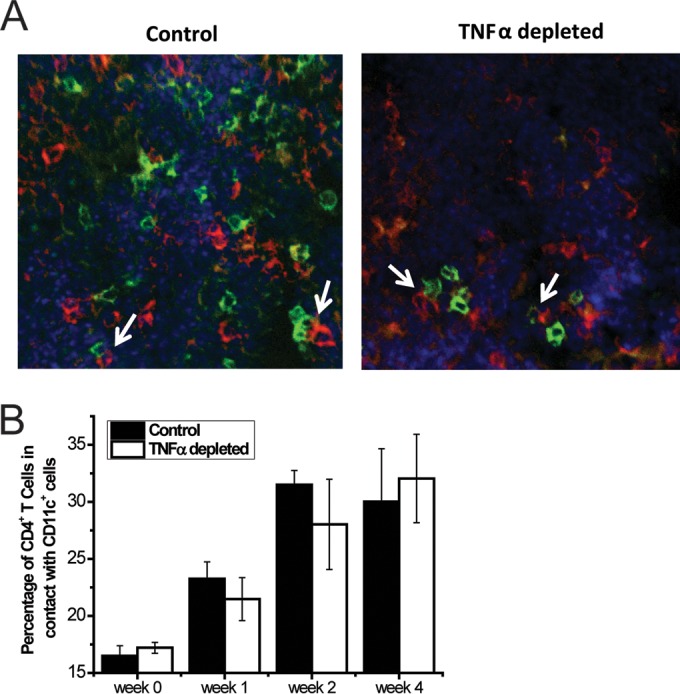
Histological examination of dendritic cell/T cell interactions in the LALN of *C. neoformans*-infected mice. (A) After isolation, LALN were frozen rapidly in OCT medium. Sections were stained with primary fluorescent antibodies against CD4 (red), CD11c (green), and DAPI (blue). The white arrows indicate pairs of CD4 and CD11c cells engaged in direct contact. Images were obtained on a fluorescence microscope, and contacts between CD4^+^ and CD11c^+^ cells were enumerated. (B) Bar graphs presenting the percentage of CD4^+^ cells in contact with CD11c^+^ cells at 0, 1, 2, and 4 wpi. Results represent means ± SEM pooled from two to three separate matched experiments (*n* ≥ 3 mice for each time point).

We next sought to determine whether early TNF-α altered the spatial relationships between DC and CD4^+^ T cells in the LALN of TNF-α-depleted mice. Specifically, we evaluated the frequency of CD4^+^ T cells in tight contact with CD11c^+^ DC in the LALN by using confocal microscopy (Fig. 5). The representative images show antibody-stained CD4^+^ T cells and CD11c^+^ cells in the LALN and the assessment of contacts between these cell subsets (Fig. 5A). Analysis of interactions between CD4^+^ and CD11c^+^ cells in the LALN revealed a comparable percentage (20 to 30%) of CD4^+^ T cells were in contact with DC in isotype control- and anti-TNF-α-treated mice (Fig. 5B). These data suggest that while early TNF-α signaling has an effect on total numbers of DC and CD4^+^ T cells in the LALN, it had no major effect on their spatial relationships and interactions.

### Early TNF-α depletion impairs classical activation and promotes alternative activation of DC in the LALN of *C. neoformans*-infected mice. 

**FIG 6:**
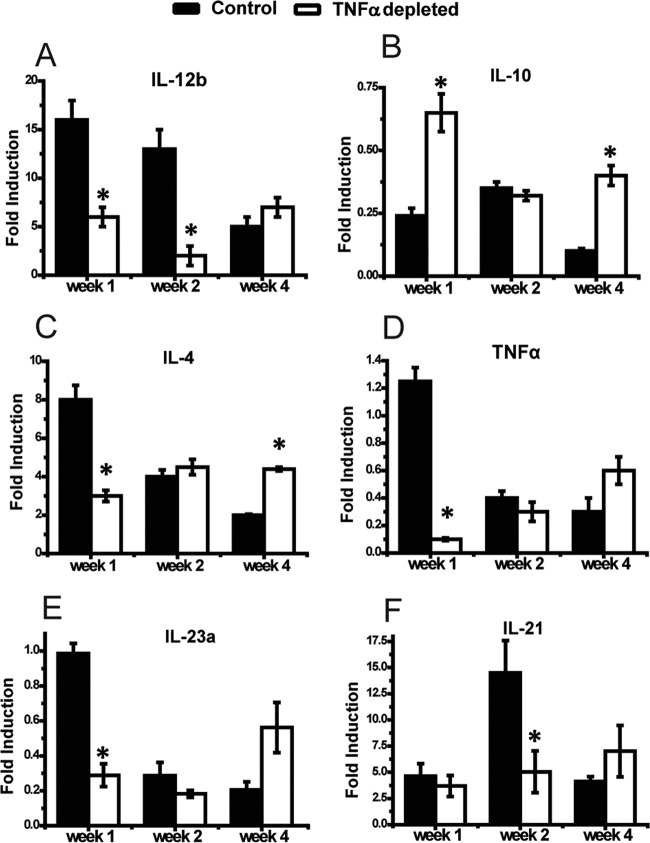
Neutralization of TNF-α suppresses mRNA expression of pro-Th1 and Th17 cytokines but promotes expression of pro-Th2 cytokines in LALN of *C. neoformans*-infected mice. DC from LALN were selected by using anti-CD11c magnetic beads. mRNA expression levels of IL-12b (A), IL-10 (B), IL-4 (C), TNF-α (D), IL-23a (E), and IL-21 (F) were analyzed by RT-qPCR. Results represent means ± SEM pooled from two to three separate matched experiments (*n* ≥ 6 mice for each time point). A two-way ANOVA with a Bonferroni posttest was used for comparison of all individual means. *, *P* < 0.05 compared with control mice.

**FIG 7:**
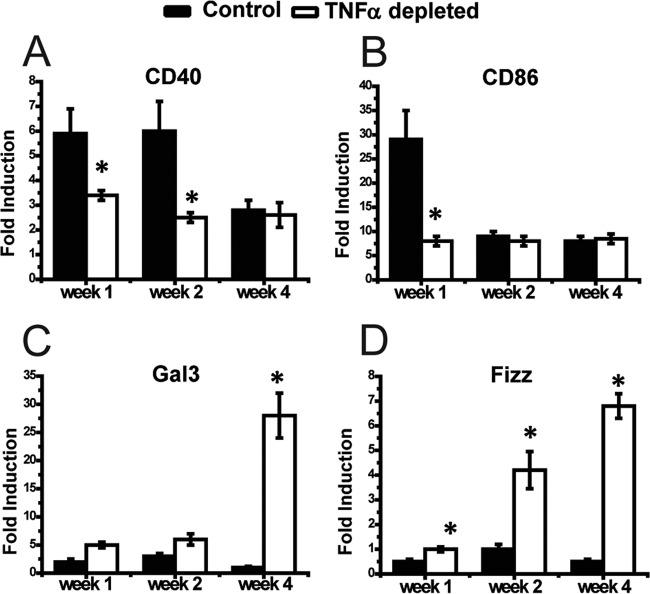
Neutralization of TNF-α suppresses mRNA expression of the DC1 signature and promotes expression of the DC2 signature in LALN of *C. neoformans*-infected mice. DC from LALN were selected using anti-CD11c magnetic beads. Classical activation of dendritic cells was analyzed by mRNA expression of signatures, such as CD40 (A) and CD86 (B). DC2 signatures, such as Gal3 (C) and Fizz (D) were also analyzed for alternative activation of dendritic cells. Results represent means ± SEM pooled from two to three separate matched experiments (*n* ≥ 6 mice for each time point). A two-way ANOVA with a Bonferroni posttest was used for comparison of all individual means. *, *P* < 0.05 compared with control mice.

The effect of TNF-α on DC polarization status in LALN during cryptococcal infection is unknown. Thus, our final objective was to determine whether changes in T cell polarization in LALN of *C. neoformans*-infected mice in response to TNF-α depletion were linked to changes in DC polarization. We first compared DC mRNA expression of phenotypic genes marking classical (DC1) and alternative (DC2) activation by using reverse transcript-quantitative PCR (RT-qPCR). Our results demonstrated that the infected control group showed a strong induction of IL-12b and profound suppression of IL-10 compared to uninfected mice, consistent with an early DC1 polarization in the LALN (Fig. 6A and B). However, we also observed a temporary elevation in IL-4 induction by LALN DC in infected control mice at 1 wpi (Fig. 6C). Importantly, TNF-α depletion resulted in a significantly lower expression level for Th1-polarizing (IL-12 and TNF-α) and Th17-polarizing (IL-23 and IL-21) cytokines, especially at 1 and/or 2 wpi compared to control mice (Fig. 6A and D to F), but the elevation in IL-4 expression in these mice at 1 wpi was not as high as that in the isotype control-treated group. However, the expression of IL-4 in TNF-α-depleted mice further increased at 2 wpi and exceeded that of control mice at 4 wpi, while expression of IL-10 was higher in TNF-α-depleted mice than in control mice at both 1 and 4 wpi (Fig. 6B and C). Reduced expression in Th1- and Th17-polarizing cytokines in DC from TNF-α-depleted mice was accompanied by lower expression of the costimulatory genes CD40 (1 and 2 wpi) and CD86 (2 wpi) (Fig. 7A and B). In contrast, DC from anti-TNF-α-treated mice showed significantly higher mRNA expression for alternative activation markers gal3 (4 wpi) and fizz1 (1, 2, and 4 wpi) than control mice (Fig. 7C and D).

**FIG 8:**
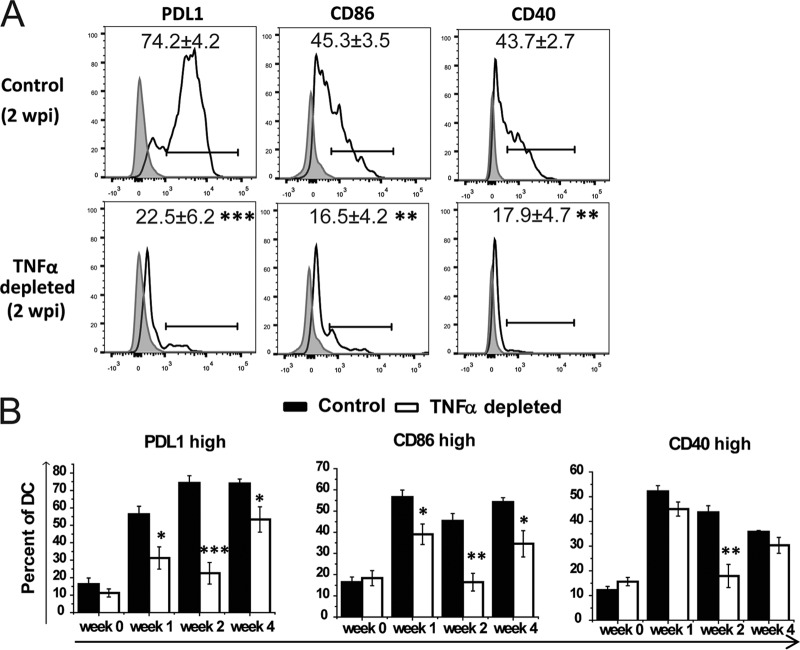
Neutralization of TNF-α suppresses surface expression of the DC1 signature in the LALN of *C. neoformans*-infected mice. Cells isolated from LALN were analyzed by flow cytometry. The surface expression levels of PDL1, CD40, and CD86 by DC (CD11c^+^ MHCII^high^) were measured. Representative histograms at 2 wpi (A) and mean fluorescence intensity of DC expressing these signatures at 0, 1, 2, and 4 wpi are shown (B). Note a significant suppression of classical activation markers by DC from LALN of TNF-α-depleted mice. Bars illustrate means ± SEM pooled from two to three separate matched experiments (*n* ≥ 6 mice for each time point). A two-way ANOVA with a Bonferroni posttest was used for comparisons of all individual means. *, *P* < 0.05; **, *P* < 0.01; ***, *P* < 0.001 compared with control mice.

Lastly, in order to exclude the interference of contaminating cells and confirm DC specificity of our qPCR data, we evaluated the surface expression of DC1 markers PDL1, CD40, and CD86 by DC from the LALN of *C. neoformans*-infected mice by using flow cytometry. In parallel with our mRNA analyses, a significantly lower proportion of DC expressing DC1 markers (PDL1 and CD86) were observed in anti-TNF-α-treated mice (relative to isotype treated controls) at 1, 2, and 4 wpi and CD40 at 2 wpi (Fig. 8). Thus, early TNF-α signaling promotes optimal activation of DC, which in light of our data is the earliest, most significant and lasting effect on DC populations in the LALN.

## DISCUSSION

While the effect of early TNF-α depletion on the development of a nonprotective immune response against *C. neoformans* infection has been demonstrated (16, 17), the immunological basis for this effects is poorly understood. Here we demonstrated that a single dose of anti-TNF-α treatment profoundly suppressed Th1 and Th17 CD4^+^ T cell responses in the lungs and induced a long-term impairment in fungal clearance. Our immunological analysis mechanistically linked these effects with a requirement for TNF-α signaling for the initial polarization of Th1 and Th17 cells at the transcriptional level and their progressive activation in LALN, which in turn is associated with optimal accumulation and classical activation of DC at this site. Collectively, our data substantially advance our understanding about the role of early TNF-α signaling in promoting protective host defenses against cryptococcal lung infection and help to explain the increased susceptibility to this pathogen in patients receiving anti-TNF-α agents.

Previous studies demonstrated that transient depletion of TNF-α during the afferent phase of the immune response to *C. neoformans* infection resulted in a dysregulated pulmonary cytokine profile (17, 18). However, those studies did not identify cellular sources of pulmonary cytokines and also showed an intriguing recovery of robust production of IFN-γ and other protective cytokines by pulmonary leukocytes at 2 wpi (18). In the current study, we have substantially advanced our understanding about the protective role of early TNF-α by showing that early TNF-α signaling is required for optimal accumulation of Th1 and Th17 cells in *C. neoformans*-infected lungs. Both IFN-γ- and IL-17A-positive CD4^+^ T cells were recruited into the lungs of control mice at 2 and 4 wpi, while transient depletion of TNF-α significantly blunted this process (Fig. 1C). The magnitudes of both Th1 and Th17 effector cytokine production by CD4^+^ T cells remained diminished at 4 wpi as a result of early TNF-α depletion (Fig. 1D and E); this effect of anti-TNF-α therapy on T cell polarization persisted even after the systemic serum levels of IFN-γ and IL-17A were no longer suppressed (Fig. 1F and G). The strong association between fungal clearance and the production of Th1/Th17 cytokines by pulmonary CD4^+^ T cells but not the overall cytokine output from pulmonary leukocytes (17, 18) provides an important insight that T cell-derived cytokines, rather than cytokines from other cellular sources, are most likely a critical determinant of successful host defense against *C. neoformans*. Collectively, these findings along with the temporal concordance support that the induction of protective Th1 and Th17 effector cells in the lungs is the most crucial aspect of TNF-α biological activity during cryptococcal infection.

T cell expansion and immune polarization are initiated in the lymph nodes as a result of interactions between antigen-specific T cells and APCs (32, 33). We showed that early TNF-α signaling supports LALN T cell expansion, orchestrates initial Th lineage polarization at the transcriptional level, and significantly contributes to the progressive CD4^+^ T cell activation in the LALN. Interestingly, these changes did not occur simultaneously. Rather, our mRNA expression data demonstrated that Th17 bias precedes the expansion of the Th1 response, as these events occurred at 1 versus 2 wpi, respectively (Fig. 2A to D). Likewise, the initial effect of TNF-α depletion on Th polarization is restricted to the Th17 arm, while Th1 parameters remain unaltered at 1 wpi (Fig. 2A to D). These results suggest that early Th17 bias in LALN may actively promote the subsequent development of Th1 immunity. This possibility is supported by other studies showing that IL-17A expression can be an upstream factor enhancing IFN-γ production during *C. neoformans* infection (34). We have shown here for the first time that this initial Th17 bias of CD4^+^ T cells in LALN is TNF-α dependent.

Importantly, a previous study showed that CD4^+^ T cells from the LALN during *C. neoformans* infection were a proliferating but nonpolarized population as determined by flow cytometry for cytokine expression (25). Our data show that CD4^+^ T cells from the LALN at least to some degree become prepolarized at the transcriptional level, as determined by mRNA analysis for both master transcriptional factors and cytokines. Together with the results of Lindell’s study (25), these data indicate that organ-specific signals from the lung tissue environment provide a final step in activation of the prepolarized Th1 and Th17 cells for cytokine production. Thus, our data provide novel insights into the polarization/activation processes occurring in the LALN which lead to the development of protective anticryptococcal immunity.

Quite surprisingly, TNF-α depletion had no effect on the mRNA expression of Th2 transcriptional regulator GATA3 and Th2 cytokines in the LALN (Fig. 2E and F). This finding was in contrast to the significant increases in systemic Th2 cytokines (Fig. 1H and I) and the reported increase in the production of Th2 cytokines by lung leukocytes post-TNF-α depletion (18). The lack of differences in GATA3 and IL-4 expression in LALN T cells obtained from anti-TNF-α-treated mice (relative to isotype control-treated mice) points out that Th2 skewing of CD4^+^ T cells may occur through the action of signals outside the LALN. In support of this view, a recent report showed that the local inflammatory environment in the lungs (infected with *C. neoformans* H99) shapes the differentiation and/or promotes the selective expansion of Th2 cells (26). It is also possible that, in the absence of strong Th1/Th17 immunity in the lungs, a Th2 response can develop by default during *C. neoformans* infection.

In addition to the changes in T cell polarization, we also observed defects in expansion and activation of CD4^+^ T cells in the lungs (data not shown) and LALN (Fig. 3C and E) of TNF-α-depleted mice. These changes in LALN corresponded to diminished numbers of CD4^+^ T cells in the lungs also may contribute to the impaired fungal clearance in TNF-α-depleted mice, in addition to the disrupted T cell polarization. Collectively, our kinetic assessment of CD4^+^ T cell populations demonstrates that disruption of early TNF-α signaling interferes with optimal expansion, initial Th1/Th17 lineage polarization events, and the overall level of CD4^+^ T cell activation in the LALN.

DC play central roles in host defenses to *C. neoformans* and other fungi through their ability to direct the development of immune responses toward either protective or nonprotective immunity (26, 35). Recruitment of sufficient numbers of DC to the LALN is one of the proposed mechanisms for ensuring development of protective immunity to *C. neoformans* (36, 37). Consistently, our data demonstrated that early TNF-α signaling is required for robust DC accumulation in the LALN, especially the CD11b^+^ DC subset, which has been shown to be particularly important for priming protective Th17 cell responses to pulmonary fungal infections (38). While early TNF-α signaling is crucial for DC accumulation and CD4^+^ T cell proliferation in the LALN, our microscopy analysis suggested that it does not affect the spatial relationships and interactions between DC/T cells in the LALN. However, the possibility cannot be excluded that the time duration of the interactions are distinct between control and TNF-α-depleted mice, which may affect subsequent T cell responses; further studies are needed to clarify this point.

The most significant defect in DC recruitment was observed at 2 wpi, while a defect in mRNA expression of the Th17-driving transcriptional factor Rorc and IL-17A is already well developed by 1 wpi. This disparity suggests that the change in DC activation, rather than diminished DC recruitment to the LALN, is the upstream cause of the immune dysregulation resulting from early TNF-α depletion. In agreement, transient TNF-α depletion has been previously associated with accumulation of immature DC (18). However, many characteristics of immature DC are also displayed by mature DC2, which motivated our broader analysis of DC1/DC2 signatures displayed by DC in the LALN. Our study revealed that expression of the DC1 signature by DC in the LALN was significantly reduced in response to TNF-α depletion (Fig. 6A and D to F and 7A and B). In contrast, expression of DC2 markers was significantly higher in TNF-α-depleted mice than in control mice (Fig. 6B and C and 7C and D). Interestingly, TNF-α depletion resulted in decreased DC1 gene expression mainly at 1 wpi, while DC2 genes showed the most significant increase at 4 wpi (Fig. 7), indicating that DC1 gene upregulation and DC2 gene downregulation are likely guided by distinct regulatory pathways, the former directly dependent on TNF-α signaling.

We showed that expression of CD40, which interestingly also belongs to the tumor necrosis factor receptor (TNFR) superfamily (39), was significantly lower in LALN DC of TNF-α-depleted mice than in isotype control-treated mice (Fig. 7A and 8). Since CD40 signaling plays protective roles during *C. neoformans* infection (40), the effects of TNF-α depletion could be mediated partly through the suppressed CD40 expression. However, the CD40 signaling pathway was shown to have a stronger effect on the magnitude of T cell responses or T cell recruitment (40), while in the present study TNF-α signaling was shown to have a stronger effect on classical DC activation and the subsequent Th1/Th17 bias during *C. neoformans* infection. Future studies explaining the cross talk between CD40 and TNFRs signaling during *C. neoformans* infection will clarify this point.

In summary, our novel findings that early TNF-α signaling is required for accumulation and classical activation of DC in the LALN provide mechanistic explanations for TNF-α-triggered Th1/Th17 CD4^+^ T cell prepolarization in LALN cells and subsequent polarization in the lung T cells in infected mice. Importantly, TNF-α-driven DC1 activation and protective T cell responses identified one mechanism of the increased susceptibility to cryptococcal and other fungal infection in patients undergoing treatments with TNF-α blockade therapies.

## MATERIALS AND METHODS

### Mice.

Female CBA/J mice were obtained from Jackson Laboratories (Bar Harbor, ME). Mice were housed under specific-pathogen-free conditions in the Animal Care Facility at the VA Ann Arbor Healthcare System. Mice were age to 8 to 10 weeks at the time of infection and were humanely euthanized by CO_2_ inhalation at the time of data collection. All experiments were approved by the University Committee on the Use and Care of Animals and the Veterans Administration Institutional Animal Care and Use Committee.

### *C. neoformans*.

*C. neoformans* strain 52D was recovered from 10% glycerol frozen stocks stored at −80°C and grown to stationary phase at 37°C in Sabouraud dextrose broth (1% Neopeptone, 2% dextrose; Difco, Detroit, MI) on a shaker. The cultures were then centrifuged and the pellets were washed with nonpyrogenic saline (Travenol, Deerfield, IL). Cells were counted via hemocytometer and diluted to 3.3 × 10^5^ yeast/ml in sterile nonpyrogenic saline.

### Intratracheal inoculation of *C. neoformans* and TNF-α depletion.

Mice were anesthetized via intraperitoneal (i.p.) injection of ketamine/xylazine (100/6.8 mg/kg of body weight) and were restrained on a foam plate. A small incision was made through the skin covering the trachea. The underlying salivary glands and muscles were separated. A 30-gauge needle was attached to a 1-ml tuberculin syringe with a *C. neoformans* suspension (3.3 × 10^5^ yeast cells/ml). Infection was performed by intratracheally injecting 30 µl (10^4^ CFU) of inoculum into the lungs. After inoculation, the skin was closed with cyanoacrylate adhesive, and the mice were monitored during recovery from the anesthesia. TNF-α depletion was performed through i.p. injection of 0.25 mg of an anti-TNF-α monoclonal antibody (MAb; clone TN3-19.12; Leinco Technologies, Inc.) or isotype control antibody (clone PIP; Leinco Technologies, Inc.) at the time of infection as described previously (17, 18). This single i.p. injection of anti-TNF-α MAb at the time of infection (day 0), as with repeated i.p. injections (day 0, 3, 6, 9), has been shown to cause a greater than 80% reduction in the TNF-α protein level in the lungs of *C. neoformans*-infected mice at 7 days p.i. (16, 18).

### Lung/spleen CFU assay.

For determination of fungal burdens in the lungs and spleens, small aliquots of dispersed lung and spleen cells were collected. Series of 10-fold dilutions of the samples were plated on Sabouraud dextrose agar plates in duplicate 10-µl aliquots and incubated at room temperature. *C. neoformans* colonies were counted 2 days later, and the number of CFU was calculated on a per-organ basis.

### Lung leukocyte isolation.

The lungs from each mouse were excised, washed in RPMI 1640, and digested enzymatically as previously described (41). In brief, lungs were minced with scissors followed by gentle magnetically activated cell sorting (MACS) homogenization and incubated at 37°C for 35 min in 5 ml/mouse digestion buffer (RPMI 1640, 5% fetal bovine serum, penicillin and streptomycin [Invitrogen, Grand Island, NY]; 1 mg/ml collagenase A [Roche Diagnostics, Indianapolis, IN]; and 30 µg/ml DNase I [Sigma, St. Louis, MO]). The cell suspension and tissue fragments were further dispersed by gentle MACS homogenization and were centrifuged. Erythrocytes in the cell pellets were lysed by addition of 3 ml NH_4_Cl buffer (0.829% NH_4_Cl, 0.1% KHCO_3_, and 0.0372% Na_2_EDTA; pH 7.4) for 3 min followed by addition of a 10-fold excess of RPMI 1640 medium. Cells were resuspended and subjected to syringe dispersion and filtered through a sterile 100-µm nylon screen (Nitex, Kansas City, MO). The filtrate was centrifuged for 30 min at 1,500 × *g* with no brake in the presence of 20% Percoll (Sigma) to separate leukocytes from cell debris and epithelial cells. Leukocyte pellets were resuspended in 5 ml complete RPMI 1640 medium and enumerated on a hemocytometer after dilution in trypan blue (Sigma).

### Total serum cytokine levels.

Serum was obtained from blood samples collected by severing the vena cava of the mice before lung excision. Blood samples were first allowed to clot and then centrifuged at 10,000 rpm for 20 min to separate serum. Quantification of serum cytokines (IFN-γ, IL-17A, IL-5, and IL-13) was performed using a LEGENDplex cytometric bead array (CBA) kit (mouse T helper cytokine panel; BioLegend, San Diego, CA) following the manufacturer’s specifications and read on an LSRII flow cytometer (Becton, Dickinson Immunocytometry Systems, Mountain View, CA). Analysis was performed using BioLegend’s LEGENDplex software.

### LALN leukocyte isolation and magnetic bead separation.

To collect LALN leukocytes, lymph nodes were removed from the mediastinum and then mechanically dispersed by using a 1-ml sterile syringe plunger to press them through a 70-µm cell strainer (BD Falcon, Bedford, MA) in complete medium. After centrifugation at 2,500 rpm for 5 min, the supernatant was removed and the cell pellets were saved for further use. In some experiments, CD4^+^ T cells of the LALN were isolated using an EasySep Mouse CD4^+^ T cell enrichment kit (Stem Cell Technologies, Vancouver, Canada). Greater than 98% of the recovered cells were CD4^+^ cells as determined using flow cytometry. CD11c^+^ DC were sorted using the EasySep mouse CD11c positive selection kit (Stem Cell Technologies). Greater than 90% of the recovered cells were CD11c^+^ as determined using flow cytometry.

### RT-qPCR.

Total RNA was prepared using TRIzol reagent (Invitrogen), and first-strand cDNA was synthesized using a reverse transcription kit (Qiagen, Hilden, Germany) according to the manufacturer’s instructions. mRNA was quantified with SYBR green-based detection by using a LightCycler96 system (Qiagen) according to the manufacturer’s protocols. Forty-five cycles of PCR (95°C for 15 s followed by 60°C for 30 s) were performed on a cDNA template. The data were normalized to 18S mRNA levels, compared with baseline expression levels in corresponding samples from the uninfected mice, and expressed as the fold induction.

### Abs and flow cytometric analysis.

For flow cytometry experiments, antibodies (Abs) were purchased from BioLegend (San Diego, CA), including rat anti-murine CD16/CD32 (clone 93); CD45 (clone 30-F11) conjugated to AF700; CD3 (clone 145-2C11), CD19 (clone 6D5), CD69 (clone H1.2F3), or IFN-γ (clone XGM1.2) conjugated to peridinin chlorophyll protein-Cy5.5; CD4 (clone GK1.5), CD11c (clone N418), or CD25 (clone DC61) conjugated to allophycocyanin-Cy7; PDL1 (clone 10F962) conjugated to allophycocyanin; and MHC class II/I-A^K^ (clone 11-5.2) conjugated to fluorescein isothiocyanate; CD103 (clone 2E7) or IL-17A (clone TC11-18H10) conjugated to phycoerythrin (PE); CD11b (clone M1/70) conjugated to PE-dazzle; CD40 (clone 3/23) conjugated to PE-Cy5; CD86 (clone GL-1) or CD45 (30-F11) conjugated to PE-Cy7; CD3 (clone 17A2) conjugated to BV650.

Ab cell staining was performed as previously described (42). Data were collected on a FACS LSR2 flow cytometer by using FACSDiva software (Becton, Dickinson Immunocytometry Systems, Mountain View, CA) and analyzed using FlowJo software (tree Star, San Carlos, CA). The following gating strategy was used to identify DC in the LALN: first, consecutive gates identified singlets, live cells, and CD45^+^ leukocytes; next, negative gating was used to remove lymphocytes (low forward scatter [FSC^low^] cells expressing CD3 or CD19). DC were then identified within the remaining cell populations as cells expressing both CD11c and high levels of MHC-II. Thereafter, the relative expression levels of PDL1, CD40, and CD86 were evaluated. In some experiments, subsets of CD11b^+^ and CD103^+^ DC were identified. To identify CD4^+^ helper T cells, singlet and live cells were first selected and the total number of CD45^+^ leukocytes were identified. Next, the CD3^+^ CD4+ cells were identified as helper T cells. Thereafter, the relative expression levels of specific cytokines or activation markers were evaluated. Total numbers of each cell population were calculated by multiplying the frequency of the population by the total number of leukocytes (e.g., the percentage of CD45^+^ cells multiplied by the original hemocytometer count of total cells). Isotype-matching control antibodies were used to set gates for positive events in all of the flow cytometric analyses.

### Confocal microscopy.

Excised LALN were rapidly frozen in OCT-filled mounting molds (Sakura Finetek USA Inc., Torrance, CA). Frozen microtome sections (6 µm thick) were mounted on adhesive slides and fixed in ice-cold acetone. Sections were washed in Dulbecco’s phosphate-buffered saline (DPBS) for 10 min and then were blocked with 10 mg/ml of LEAF-purified anti-mouse CD16/32 antibody (BioLegend) in DPBS for 30 min at room temperature. Primary antibodies were added at a 1:400 or 1:1,000 (vol/vol) dilution in DPBS for Alexa Fluor 488 anti-mouse CD11c antibody or Alexa Fluor 594 anti-mouse CD4 antibody, respectively. Slides were then incubated for 1 h in the dark at room temperature. After washing in DPBS three times (3 min per wash), ProLong Gold antifade mountant with 4′,6-diamidino-2-phenylindole (DAPI; Life Technologies) was used for mounting the coverslips. In control experiments, matching isotype antibodies (isotype and fluorochrome) were used instead of the primary antibodies.

Confocal analysis was performed using a spinning disk confocal microscope (Olympus, America Inc., Center Valley, PA) with a digital charge-coupled-device camera (Hamamatsu Photonics, Hamamatsu, Japan) for image capture and an arc lamp illumination source providing excitation wavelengths of 350 to 700 nm and three-color emission analyses. The acquired digital images were processed and analyzed using Stereo Investigator software version 9 (MBF Bioscience, Williston, VT).

For interaction analysis, images were captured at ×200 magnification. DC were defined as cells expressing high-threshold CD11c fluorescence and helper T cells were defined as cells expressing CD4 fluorescence. For cell contact analysis, the total number of close contacts between CD11c^+^ cells and CD4^+^ cells was determined and normalized to the total number of identified CD4^+^ T cells. At least 10 fields were randomly sampled per mouse.

### Calculations and statistics.

All values are reported as means ± standard errors of the means (SEM). A two-way analysis of variance (ANOVA) with a Bonferroni posttest was used for comparison of all individual means. Means with *P* values of <0.05 were considered significantly different.

## References

[1] Bradley JR 2008 TNFα mediated inflammatory disease. J Pathol 214:149–160. doi:10.1002/path.2287. 18161752

[2] Tsiodras S, Samonis G, Boumpas DT, Kontoyiannis DP 2008 Fungal infections complicating tumor necrosis factor α blockade therapy. Mayo Clin Proc 83:181–194. doi:10.1016/S0025-6196(11)60839-2. 18241628

[3] Ellerin T, Rubin RH, Weinblatt ME 2003 Infections and anti–tumor necrosis factor α therapy. Arthritis Rheum 48:3013–3022. doi:10.1002/art.11301. 14613261

[4] Rohatgi S, Pirofski LA 2015 Host immunity to *Cryptococcus neoformans*. Future Microbiol 10:565–581. doi:10.2217/fmb.14.132. 25865194 PMC4523559

[5] Durden FM, Elewski B 1997 Fungal infections in HIV-infected patients. Semin Cutan Med Surg 16:200–212. doi:10.1016/S1085-5629(97)80043-0. 9300631

[6] Chuck SL, Sande MA 1989 Infections with *Cryptococcus neoformans* in the acquired immunodeficiency syndrome. N Engl J Med 321:794–799. doi:10.1056/NEJM198909213211205. 2671735

[7] Pappas PG, Perfect JR, Cloud GA, Larsen RA, Pankey GA, Lancaster DJ, Henderson H, Kauffman CA, Haas DW, Saccente M, Hamill RJ, Holloway MS, Warren RM, Dismukes WE 2001 Cryptococcosis in human immunodeficiency virus-negative patients in the era of effective azole therapy. Clin Infect Dis 33:690–699. doi:10.1086/322597. 11477526

[8] Olszewski MA, Zhang Y, Huffnagle GB 2010 Mechanisms of cryptococcal virulence and persistence. Future Microbiol 5:1269–1288. doi:10.2217/fmb.10.93. 20722603

[9] Wager CML, Wormley FL Jr 2015 Is development of a vaccine against *Cryptococcus neoformans* feasible? PLoS Pathog 11:e1004843. doi:10.1371/journal.ppat.1004843. 26087178 PMC4472670

[10] He X, Lyons DM, Toffaletti DL, Wang F, Qiu Y, Davis MJ, Meister DL, Dayrit JK, Lee A, Osterholzer JJ, Perfect JR, Olszewski MA 2012 Virulence factors identified by *Cryptococcus neoformans* mutant screen differentially modulate lung immune responses and brain dissemination. Am J Pathol 181:1356–1366. doi:10.1016/j.ajpath.2012.06.012. 22846723 PMC3463625

[11] Osterholzer JJ, Surana R, Milam JE, Montano GT, Chen GH, Sonstein J, Curtis JL, Huffnagle GB, Toews GB, Olszewski MA 2009 Cryptococcal urease promotes the accumulation of immature dendritic cells and a non-protective T2 immune response within the lung. Am J Pathol 174:932–943. doi:10.2353/ajpath.2009.080673. 19218345 PMC2665753

[12] Zhang Y, Wang F, Tompkins KC, McNamara A, Jain AV, Moore BB, Toews GB, Huffnagle GB, Olszewski MA 2009 Robust Th1 and Th17 immunity supports pulmonary clearance but cannot prevent systemic dissemination of highly virulent *Cryptococcus neoformans* H99. Am J Pathol 175:2489–2500. doi:10.2353/ajpath.2009.090530. 19893050 PMC2789623

[13] Kawakami K, Hossain Qureshi M, Zhang T, Koguchi Y, Xie Q, Kurimoto M, Saito A 1999 Interleukin-4 weakens host resistance to pulmonary and disseminated cryptococcal infection caused by combined treatment with interferon-γ-inducing cytokines. Cell Immunol 197:55–61. doi:10.1006/cimm.1999.1557. 10555996

[14] Müller U, Stenzel W, Köhler G, Werner C, Polte T, Hansen G, Schütze N, Straubinger RK, Blessing M, McKenzie AN, Brombacher F, Alber G 2007 IL-13 induces disease-promoting type 2 cytokines, alternatively activated macrophages and allergic inflammation during pulmonary infection of mice with *Cryptococcus neoformans*. J Immunol 179:5367–5377. doi:10.4049/jimmunol.179.8.5367. 17911623

[15] Blackstock R, Murphy JW 2004 Role of interleukin-4 in resistance to *Cryptococcus neoformans* infection. Am J Respir Cell Mol Biol 30:109–117. doi:10.1165/rcmb.2003-0156OC. 12855407

[16] Huffnagle GB, Toews GB, Burdick MD, Boyd MB, McAllister KS, McDonald RA, Kunkel SL, Strieter RM 1996 Afferent phase production of TNF-alpha is required for the development of protective T cell immunity to *Cryptococcus neoformans*. J Immunol 157:4529–4536. PubMed. 8906831

[17] Herring AC, Lee J, McDonald RA, Toews GB, Huffnagle GB 2002 Induction of interleukin-12 and gamma interferon requires tumor necrosis factor alpha for protective T1-cell-mediated immunity to pulmonary *Cryptococcus neoformans* infection. Infect Immun 70:2959–2964. doi:10.1128/IAI.70.6.2959-2964.2002. 12010985 PMC127967

[18] Herring AC, Falkowski NR, Chen G.-H, McDonald RA, Toews GB, Huffnagle GB 2005 Transient neutralization of tumor necrosis factor alpha can produce a chronic fungal infection in an immunocompetent host: potential role of immature dendritic cells. Infect Immun 73:39–49. doi:10.1128/IAI.73.1.39-49.2005. 15618139 PMC538928

[19] Banchereau J, Steinman RM 1998 Dendritic cells and the control of immunity. Nature 392:245–252. doi:10.1038/32588. 9521319

[20] Bozza S, Gaziano R, Spreca A, Bacci A, Montagnoli C, di Francesco P, Romani L 2002 Dendritic cells transport conidia and hyphae of *Aspergillus fumigatus* from the airways to the draining lymph nodes and initiate disparate Th responses to the fungus. J Immunol 168:1362–1371. doi:10.4049/jimmunol.168.3.1362. 11801677

[21] Cook PC, Jones LH, Jenkins SJ, Wynn TA, Allen JE, MacDonald AS 2012 Alternatively activated dendritic cells regulate CD4+ T-cell polarization in vitro and in vivo. Proc Natl Acad Sci U S A 109:9977–9982. doi:10.1073/pnas.1121231109. 22660926 PMC3382483

[22] Weitnauer M, Schmidt L, Ng Kuet Leong N, Muenchau S, Lasitschka F, Eckstein V, Hübner S, Tuckermann J, Dalpke AH 2014 Bronchial epithelial cells induce alternatively activated dendritic cells dependent on glucocorticoid receptor signaling. J Immunol 193:1475–1484. doi:10.4049/jimmunol.1400446. 24965772

[23] Eastman AJ, Osterholzer JJ, Olszewski MA 2015 Role of dendritic cell-pathogen interactions in the immune response to pulmonary cryptococcal infection. Future Microbiol 10:1837–1857. doi:10.2217/fmb.15.92. 26597428 PMC5720351

[24] Fairweather D, Cihakova D 2009 Alternatively activated macrophages in infection and autoimmunity. J Autoimmun 33:222–230. doi:10.1016/j.jaut.2009.09.012. 19819674 PMC2783278

[25] Lindell DM, Moore TA, McDonald RA, Toews GB, Huffnagle GB 2006 Distinct compartmentalization of CD4+ T-cell effector function versus proliferative capacity during pulmonary cryptococcosis. Am J Pathol 168:847–855. doi:10.2353/ajpath.2006.050522. 16507900 PMC1606518

[26] Wiesner DL, Specht CA, Lee CK, Smith KD, Mukaremera L, Lee ST, Lee CG, Elias JA, Nielsen JN, Boulware DR, Bohjanen PR, Jenkins MK, Levitz SM, Nielsen K 2015 Chitin recognition via chitotriosidase promotes pathologic type-2 helper T cell responses to cryptococcal infection. PLoS Pathog 11:e1004701. doi:10.1371/journal.ppat.1004701. 25764512 PMC4357429

[27] Wozniak KL, Vyas JM, Levitz SM 2006 In vivo role of dendritic cells in a murine model of pulmonary cryptococcosis. Infect Immun 74:3817–3824. doi:10.1128/IAI.00317-06. 16790753 PMC1489690

[28] Ramirez-Ortiz ZG, Means TK 2012 The role of dendritic cells in the innate recognition of pathogenic fungi (*A. fumigatus*, *C. neoformans* and *C. albicans*). Virulence 3:635–646. doi:10.4161/viru.22295. 23076328 PMC3545945

[29] D’Ostiani CF, Del Sero G, Bacci A, Montagnoli C, Spreca A, Mencacci A, Ricciardi-Castagnoli P, Romani L 2000 Dendritic cells discriminate between yeasts and hyphae of the fungus *Candida albicans.* Implications for initiation of T helper cell immunity *in vitro* and *in vivo*. J Exp Med 191:1661–1674. doi:10.1084/jem.191.10.1661. 10811860 PMC2193147

[30] Guilliams M, Lambrecht BN, Hammad H 2013 Division of labor between lung dendritic cells and macrophages in the defense against pulmonary infections. Mucosal Immunol 6:464–473. doi:10.1038/mi.2013.14. 23549447

[31] Del Rio ML, Bernhardt G, Rodriguez-Barbosa JI, Förster R 2010 Development and functional specialization of CD103+ dendritic cells. Immunol Rev 234:268–281. doi:10.1111/j.0105-2896.2009.00874.x. 20193025

[32] Friedl P, Den Boer AT, Gunzer M 2005 Tuning immune responses: diversity and adaptation of the immunological synapse. Nat Rev Immunol 5:532–545. doi:10.1038/nri1647. 15999094

[33] Hommel M 2004 On the dynamics of T-cell activation in lymph nodes. Immunol Cell Biol 82:62–66. doi:10.1111/j.1440-1711.2004.01209.x. 14984596

[34] Murdock BJ, Huffnagle GB, Olszewski MA, Osterholzer JJ 2014 Interleukin-17A enhances host defense against cryptococcal lung infection through effects mediated by leukocyte recruitment, activation, and gamma interferon production. Infect Immun 82:937–948. doi:10.1128/IAI.01477-13. 24324191 PMC3957981

[35] Bauman SK, Nichols KL, Murphy JW 2000 Dendritic cells in the induction of protective and nonprotective anticryptococcal cell-mediated immune responses. J Immunol 165:158–167. doi:10.4049/jimmunol.165.1.158. 10861048

[36] Bauman SK, Huffnagle GB, Murphy JW 2003 Effects of tumor necrosis factor alpha on dendritic cell accumulation in lymph nodes draining the immunization site and the impact on the anticryptococcal cell-mediated immune response. Infect Immun 71:68–74. doi:10.1128/IAI.71.1.68-74.2003. 12496150 PMC143367

[37] Traynor TR, Herring AC, Dorf ME, Kuziel WA, Toews GB, Huffnagle GB 2002 Differential roles of CC chemokine ligand 2/monocyte chemotactic protein-1 and CCR2 in the development of T1 immunity. J Immunol 168:4659–4666. doi:10.4049/jimmunol.168.9.4659. 11971015

[38] Schlitzer A, McGovern N, Teo P, Zelante T, Atarashi K, Low D, Ho AW, See P, Shin A, Wasan PS, Hoeffel G, Malleret B, Heiseke A, Chew S, Jardine L, Purvis HA, Hilkens CM, Tam J, Poidinger M, Stanley ER 2013 IRF4 transcription factor-dependent CD11b+ dendritic cells in human and mouse control mucosal IL-17 cytokine responses. Immunity 38:970–983. doi:10.1016/j.immuni.2013.04.011. 23706669 PMC3666057

[39] Elgueta R, Benson MJ, De Vries VC, Wasiuk A, Guo Y, Noelle RJ 2009 Molecular mechanism and function of CD40/CD40L engagement in the immune system. Immunol Rev 229:152–172. doi:10.1111/j.1600-065X.2009.00782.x. 19426221 PMC3826168

[40] Chen G.-H, Osterholzer JJ, Choe MY, McDonald RA, Olszewski MA, Huffnagle GB, Toews GB 2010 Dual roles of CD40 on microbial containment and the development of immunopathology in response to persistent fungal infection in the lung. Am J Pathol 177:2459–2471. doi:10.2353/ajpath.2010.100141. 20864680 PMC2966803

[41] Olszewski MA, Huffnagle GB, McDonald RA, Lindell DM, Moore BB, Cook DN, Toews GB 2000 The role of macrophage inflammatory protein-1 alpha/CCL3 in regulation of T cell-mediated immunity to *Cryptococcus neoformans* infection. J Immunol 165:6429–6436. doi:10.4049/jimmunol.165.11.6429. 11086082

[42] Qiu Y, Dayrit JK, Davis MJ, Carolan JF, Osterholzer JJ, Curtis JL, Olszewski MA 2013 Scavenger receptor A modulates the immune response to pulmonary *Cryptococcus neoformans* infection. J Immunol 191:238–248. doi:10.4049/jimmunol.1203435. 23733871 PMC4007509

